# MicroBVS: Dirichlet-tree multinomial regression models with Bayesian variable selection - an R package

**DOI:** 10.1186/s12859-020-03640-0

**Published:** 2020-07-22

**Authors:** Matthew D. Koslovsky, Marina Vannucci

**Affiliations:** Department of Statistics, Rice University, Houston, TX USA

**Keywords:** Bayesian analysis, Compositional data, Dirichlet-tree multinomial regression, Microbiome, Variable selection

## Abstract

**Background:**

Understanding the relation between the human microbiome and modulating factors, such as diet, may help researchers design intervention strategies that promote and maintain healthy microbial communities. Numerous analytical tools are available to help identify these relations, oftentimes via automated variable selection methods. However, available tools frequently ignore evolutionary relations among microbial taxa, potential relations between modulating factors, as well as model selection uncertainty.

**Results:**

We present MicroBVS, an R package for Dirichlet-tree multinomial models with Bayesian variable selection, for the identification of covariates associated with microbial taxa abundance data. The underlying Bayesian model accommodates phylogenetic structure in the abundance data and various parameterizations of covariates’ prior probabilities of inclusion.

**Conclusion:**

While developed to study the human microbiome, our software can be employed in various research applications, where the aim is to generate insights into the relations between a set of covariates and compositional data with or without a known tree-like structure.

## Background

The human microbiome is a collection of prokaryotes, archaea, fungi, and viruses which may vary in composition depending on an individual’s health, diet, and environment [1, 2]. High-throughput sequencing technologies enable researchers to characterize the composition of the microbiome by quantifying richness, diversity, and abundances (see [2] for a detailed review). Characterization of the microbiome is especially critical to the study of chronic diseases such as cancer and diabetes that may be associated with key changes in the microbiome [2].

Models developed to investigate microbial taxa abundance data collected on the human microbiome must be able to handle numerous analytical challenges observed in practice, including overdispersion, complex correlation structures, sparsity, high-dimensionality, and known biological information [2]. Recently, the Dirichlet-multinomial (DM) distribution has been used to model microbial count data, since it can accommodate overdispersion induced by sample heterogeneity and varying proportions among samples [3–6]. However, the DM model only assumes that counts are negatively correlated. Alternatively, the Dirichlet-tree multinomial model (DTM) inherits the DM’s ability to handle overdispersed data and can model general correlation structures between counts as well as naturally incorporate structural information [7, 8]. Microbial abundance data, in particular, have been shown to depend on the evolutionary relations among taxa represented by a phylogenetic tree [9–11].

An important question in human microbiome research is to identify associations between microbial abundance data and clinical covariates, such as KEGG orthology pathways or dietary intake [5, 6, 9, 12–16]. For this, researchers often use penalized likelihood methods to simultaneously estimate regression coefficients and select covariates [6, 9]. These models are typically quite efficient and have shown good predictive accuracy [6, 9]. However, the ability of these models to incorporate information about known relations between covariates is limited due to the requirement of complex optimization routines [9]. Additionally, they do not accommodate model selection uncertainty while performing selection.

Alternatively, Bayesian variable selection methods are able to accommodate complex, high-dimensional data structures and fully account for model uncertainty over covariate selection [17, 18]. A common approach for Bayesian variable selection is to employ a spike-and-slab prior for regression coefficients that depends on a latent inclusion indicator for each covariate [18]. In this model formulation, unassociated covariates are pushed out of the model and associated covariates’ regression coefficients are freely estimated. Recently, Wadsworth et al. [5] developed an approach for identifying KEGG orthology pathways that were associated with multivariate count data using a DM regression model with spike-and-slab priors. Through simulations, they demonstrate improved performance of their method on selecting covariates when compared to alternative methods, including the penalized likelihood approach of [6].

We present MicroBVS, an R package for Dirichlet-tree multinomial models with Bayesian variable selection, for the identification of covariates associated with microbial taxa abundance data. The underlying Bayesian model extends the work of Wadsworth et al. [5] by accommodating tree-like structure between the compositional data and also includes various parameterizations of covariates’ prior probabilities of inclusion. While developed to study the human microbiome, our software can be employed in various research applications, where the aim is to generate insights into the relations between a set of covariates and compositional data with or without a known tree-like structure.

## Implementation

### Software implementation

Our contributed R package provides a general approach for identifying covariates associated with compositional data. At the core is a Markov chain Monte Carlo (MCMC) algorithm that generates posterior samples of model parameters for inference. The MCMC algorithm is written in C++ to increase performance time and accessed through R wrapper functions using Rcpp and RcppArmadillo [19, 20]. The package extends the work of Wadsworth et al. [5] by accommodating tree-like structure between the compositional data via a DTM regression model. As a result, our approach incorporates the contributions of [5] as a special case and additionally is flexible to various prior probability of inclusion parameterizations. The package has built-in functionality to simulate data in user-specified research scenarios to assess selection performance and conduct sensitivity analyses. Additionally, various auxiliary R functions are incorporated to help researchers assess convergence, draw inference from the MCMC samples, and plot results. The package includes a vignette with worked examples using simulated data.

### Data input and output

While designed to study microbial abundance data, our package can handle any research setting aimed at identifying factors associated with compositional data. Thus in microbiome analyses, our package is agnostic to the sequencing approach used to quantify microbial samples. In addition to compositional data, the method requires a set of covariates collected for each subject and a tree object that can be read by the R package ape [21]. Before analysis, we recommend standardizing continuous covariates and reparameterizing categorical covariates using indicator variables. Standard for any Bayesian approach, our algorithm requires the specification of various hyperparameters in the model. While we have set default values for each of the hyperparameters, the vignette contains details of their function in the algorithm as well as recommendations for their adjustment. Technical details of the model can be found in the Supplementary Material.

Once the algorithm has run, a list of MCMC samples for each of the parameters’ posterior distributions is outputted. This list includes MCMC samples for intercept terms, covariates’ respective regression coefficients, and latent inclusion indicators for covariates, which take on values of zero or one, corresponding to exclusion or inclusion in the model. Inclusion in the model is determined if the marginal posterior probability of inclusion (MPPI), calculated as the average of the MCMC inclusion indicator samples for each covariate-branch combination, is ≥0.50 [22]. An alternative inclusion threshold can be obtained using a Bayesian false discovery rate, which controls for multiplicity [23]. In addition to the functions provided in the package to draw posterior inference, the output can easily be transformed into a format that is readable by the coda package in R for further summaries, plotting, and diagnostics [24].

## Application

To demonstrate the functionality of our software, we apply it to a benchmark data set collected to study the relation between dietary intake and the human gut microbiome [15]. Previously, Wang and Zhao [9] proposed a penalized DTM regression model to identify dietary intake covariates associated with genus-level operational taxonomic units (OTUs) on a subset of these data. For comparison, we apply our software to the same data. Briefly, the data used in this analysis consist of 28 genera-level OTU counts obtained from 16S rRNA sequencing and a corresponding set of 97 dietary intake covariates derived from diet information collected using a food frequency questionnaire on 98 subjects.

**Fig. 1:**
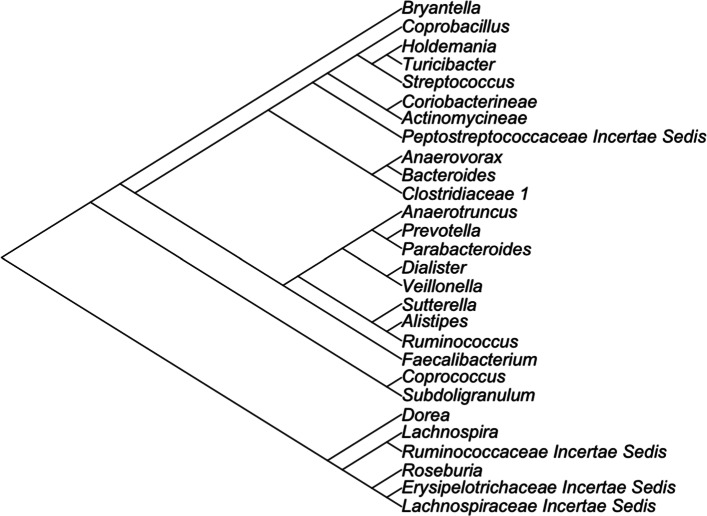
Phyologentic tree for application data

In this analysis, the model was run on these data using a DTM regression model. The phylogentic tree used in this analysis is presented in Fig. 1. We assumed a non-informative Beta-Binomial prior for inclusion indicators (*a*=*b*=1). The MCMC algorithm was run for 150,000 iterations. After a burn-in of 75,000 samples, inference was drawn from the remaining 75,000. Visual inspection of the trace plots for the number of active covariates in the model and the log posterior distribution indicated good convergence and mixing. A covariate’s inclusion in the model was determined using a Bayesian false discovery rate of 0.01, corresponding to a MPPI ≥0.89. Additionally, we ran the method of [9] with penalty parameter *γ*=0.25, corresponding to a sparse grouped lasso prior, over a grid of *λ* values, similar to their analysis. For the penalized approach, the best model was then chosen by minimizing the Akaike information criterion [25].

1 $$\begin{array}{*{20}l} {Y}_{ijk}=\mu+{Tri}+{T}_{j}+{A}_{k}+{e}_{ijk} \end{array} $$

## Results and discussion

**Fig. 2:**
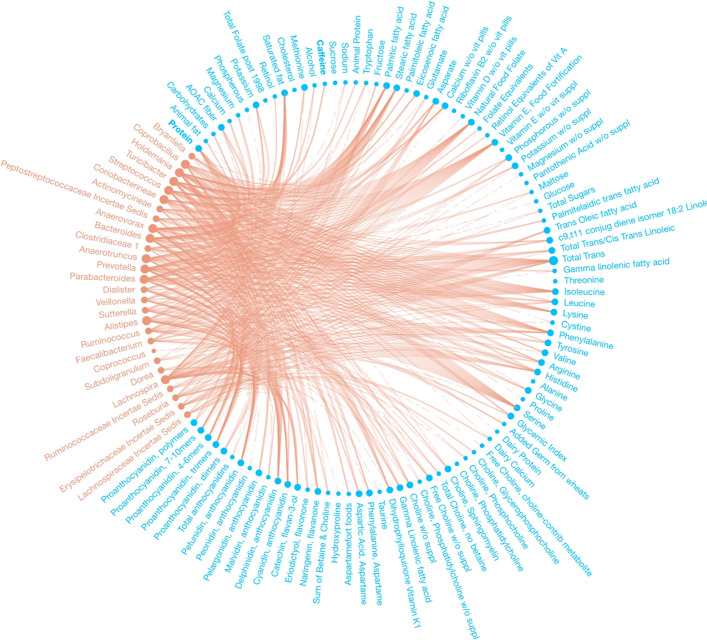
Network of associations found using the proposed DTM MCMC algorithm. Identified associations are represented by edges between microbial taxa (red) and dietary factors (blue)

**Fig. 3:**
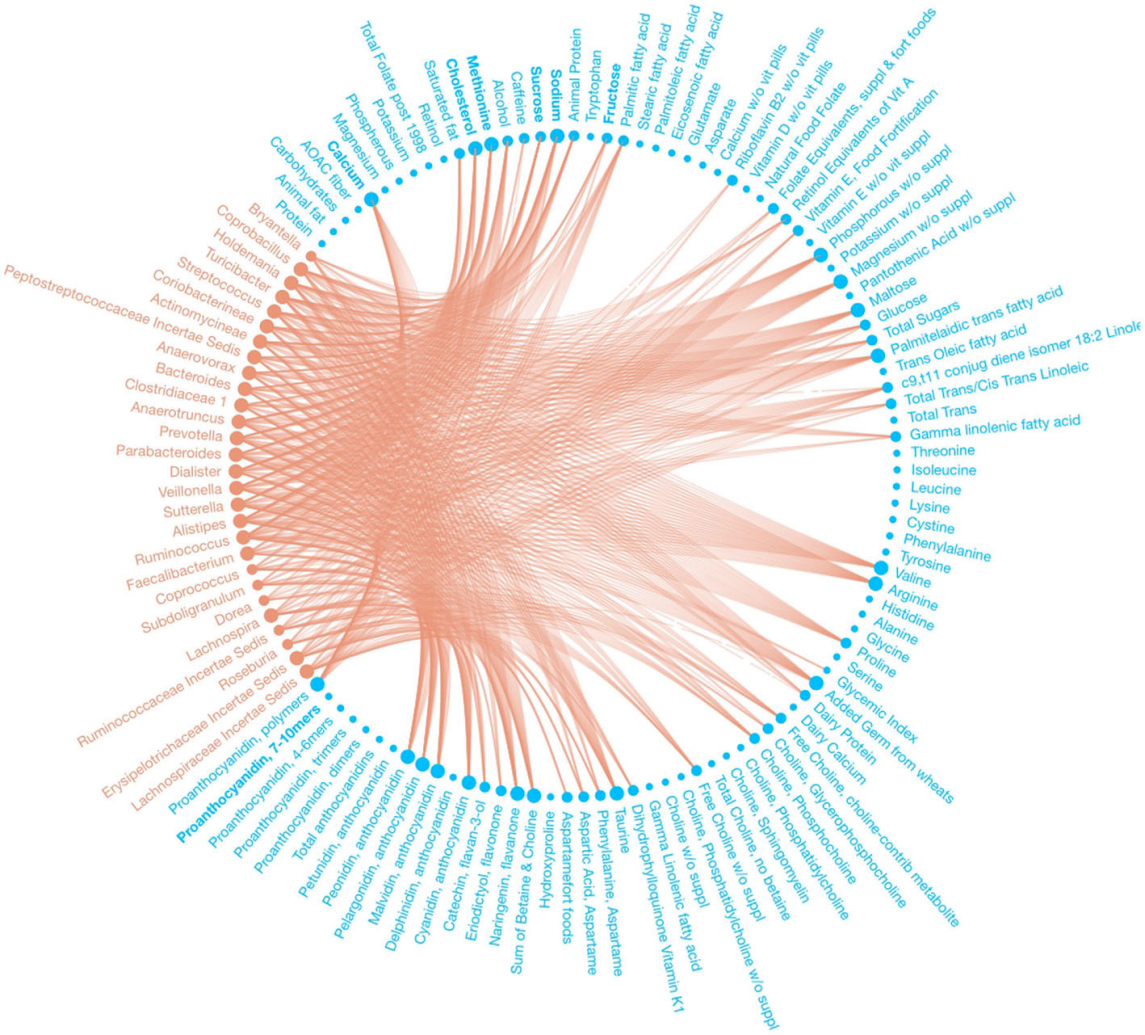
Network of associations found using the method of [9]. Identified associations are represented by edges between microbial taxa (red) and dietary factors (blue)

**Fig. 4:**
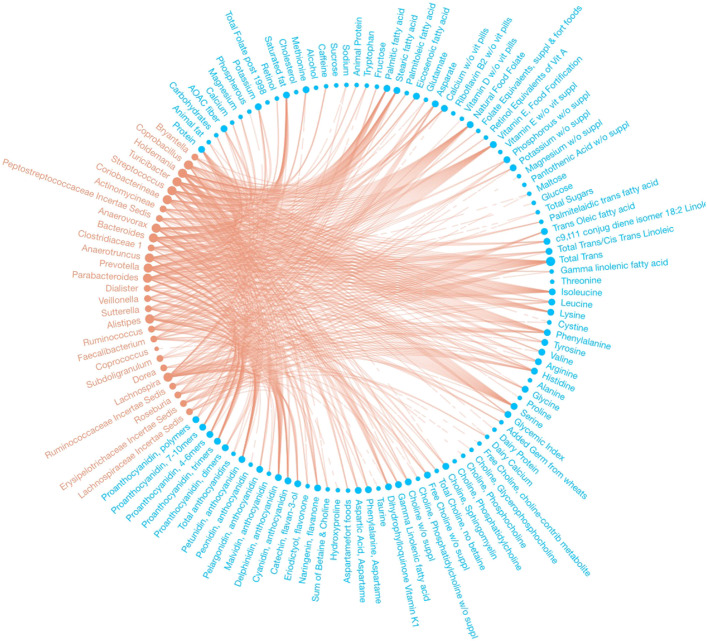
Network of associations found using the proposed DTM MCMC algorithm and not the method of [9]. Identified associations are represented by edges between microbial taxa (red) and dietary factors (blue)

We identified 232 dietary factor-branch associations with our Bayesian variable selection method for DTM regression models, whereas the penalized approach identified 271 associations overall. See Figs. 2 and 3 for a network representation of the associations identified by each model. Figure 4 captures the associations that our proposed method found that the penalized approach excluded. We observed that the penalized approach tended to identify similar dietary factors across taxa. These results may reflect the structure imposed by the sparse grouped lasso penalty used in the penalized approach. While the Beta-Binomial prior for inclusion indicators does not impose any structural relations between covariates, the MicroBVS package can be specified with graph-based inclusion priors, similar to [26, 27]. See the vignette for details regarding inclusion indicator prior specification.

**Fig. 5:**
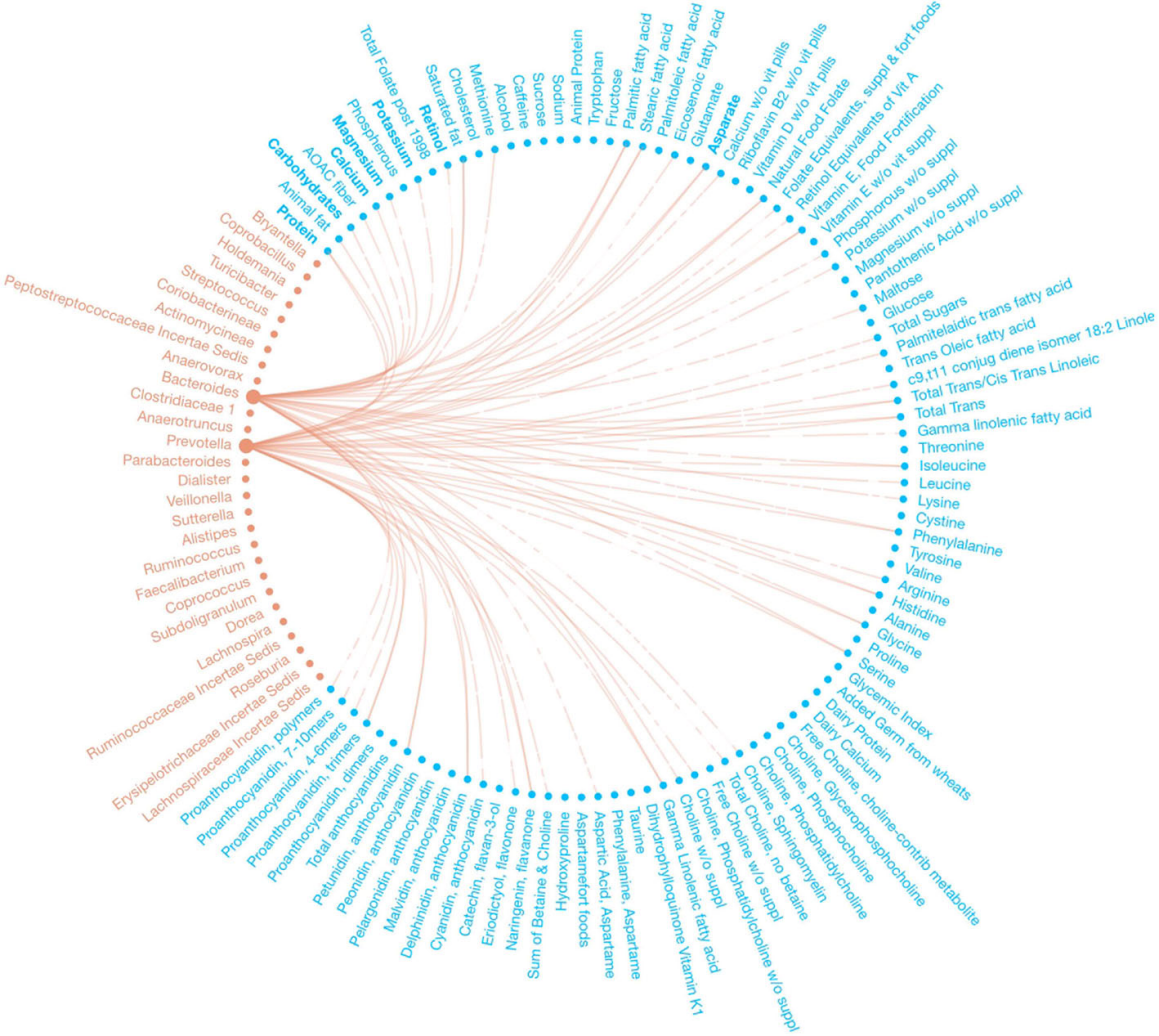
Network of associations found for Bacteroides and Prevotella using the proposed DTM MCMC algorithm and not the method of [9]. Identified associations are represented by edges between microbial taxa (red) and dietary factors (blue)

Similar to our approach, Wang and Zhao’s method identified factors associated with each branch of the phylogenetic tree. To summarize association results at the genus-level, they reported the most frequently selected dietary intake covariates along the paths from the root node of the phylogenetic tree to the leaf nodes representing two genera previously used to define enterotypes of the human microbiome [15, 28], Bacteroides and Prevotella, across 100 randomly split testing and training data sets. For comparison, we present a network graph of the dietary intake covariates identified by our model, but not the method of [9], along these same paths using the full data set (Fig. 5).

As in Wu et al. [15], we found associations between Bacteroides and various amino acids and fatty acids. Relations between amino acids and Bacteroides were also confirmed in [9]. Both [9] and [15] found Prevotella to be associated with a carbohydrate-based diet. Similar to [9], we identified *Naringenin, flavanone* and *Total Trans/Cis Trans Linoleic* as associated with Prevotella. Additionally, we identified relations between Prevotella and *Methionine*, *Phenylalanine*, *Total Choline, no betaine*, and *Sum of Betaine and Choline*, similar to [15]. Compared to [6], who proposed a penalized likelihood approach for a DM model, we also found relations between Bacteroides and *Animal fat, Eriodictyol, flavonone*, and *Maltose* as well as between Prevotella and *Choline, Phosphatidylcholine*.

Bayesian variable selection methods for regression models have shown better selection performance than penalized approaches [5, 29, 30]. However, these approaches are typically computationally less efficient. For the DTM regression models of this paper, the dimension of the model space grows dramatically as a function of the number of covariates, number of leaf (or root) nodes, and complexity of the phylogenetic tree. Specifically for *B* branches and *P* covariates, there are 2^*B*×*P*^ potential models to choose from. In addition to large parameter spaces, convergence of the model is highly dependent on the correlation structure between covariates and count data, as well as the sparsity level of the model. For the analysis of this paper, the DTM model took around 9 hours to run 150,000 iterations on a 2.5 GHz dual-core Intel Core i5 processor with 8 GB RAM. To maintain reasonable computation times and selection performance, we recommend applying the Bayesian DTM model to small-to-medium sized microbiome data sets, that is, with less than 100 compositional components and moderate-to-large tree-structures when *B*×*P*>>*n*. Larger data sets might be analyzed by employing the Dirichlet-multinomial regression model of Wadsworth et al. [5], which does not incorporate the phylogenetic tree. This option is available within the MicroBVS software.

Our software implementation includes some of the most commonly used inclusion indicator priors. In practice, researchers are often interested in identifying higher-order terms, such as interactions, or grouped covariates. Future developments of the software may include functionality to handle these type of settings following [31]. Additionally, we assume that all of the covariate relations in the model are linear, which may not be realistic. Alternative priors for regression coefficients are available that can handle non-parametric relations (e.g., Dirichlet process priors). As the dimension of the model grows, inference becomes challenging. In addition to the posterior inference tools we provide in this version of the R package, more advanced visualization tools may permit a deeper understanding of the model’s results in applications. While using a fully Bayesian MCMC algorithm for posterior inference accommodates both parameter estimation and model selection uncertainty, our approach may not scale as well as approximate Bayesian methods, which may underestimate model uncertainty, to extremely large data sets. For DM and negative binomial regression models, [32] devised an efficient, variational Bayes variable selection approach via spike-and-slab priors. In future work, we aim to incorporate a variational alternative for DTM regression models, as well as extend our package to handle other data structures commonly found in microbiome research (e.g., zero-inflated counts, negative binomial distributions).

## Conclusions

This software package provides a general Bayesian approach for identifying factors associated with compositional data that may have known tree-like structure. Additionally, the package is accompanied by a detailed vignette that contains a step-by-step tutorial demonstrating how to use the package in practice. Together, our user-friendly package enables researchers to investigate heterogeneity in compositional data potentially explained by a set of covariates. While we demonstrate our package in the context of human microbiome data, it can be applied to various research settings.

### Availability of data and requirements

Project name: MicroBVS Project home page: https://github.com/mkoslovsky/MicroBVS Operating system(s): Linux, Mac OS, Windows Programming language: R and C++ Other requirements: R Rcpp RcppArmadillo ape MCMCpack mvtnorm ggplot2 GGMselect devtools ape igraphLicense: MITAny restrictions to use by non-academics: None. Data Availability: All simulated data can be generated using the R package. Data analyzed in the Case Study are available in the R package [15].

## Supplementary information

**Additional file 1** Supplementary Material for MicroBVS: Dirichlet-Tree Multinomial Regression Models with Bayesian Variable Selection - an R Package.

## Abbreviations

DM: Dirichlet-multinomial
DTM: Dirichlet-tree multinomial
GB: Gigabyte
GHz: Gigahertz
KEGG: Kyoto encyclopedia of genes and genomes
MCMC: Markov chain Monte Carlo
MPPI: Marginal posterior probability of inclusion
OTU: Operational taxonomic unit
RAM: Random access memory
