# Correction to: MicroBVS: Dirichlet-tree multinomial regression models with Bayesian variable selection - an R package

**DOI:** 10.1186/s12859-020-03912-9

**Published:** 2020-12-28

**Authors:** Matthew D. Koslovsky, Marina Vannucci

**Affiliations:** Department of Statistics, Rice University, Houston, TX USA

## Correction to: BMC Bioinformatics (2020) 21:301 BMC 10.1186/s12859-020-03640-0

Following the publication of the original article [1], it was noted that due to a typesetting error Eq. 1 was placed incorrectly.

1 $${\text{Yijk }} = \upmu + {\text{Tri}} + {\text{Tj}} + {\text{Ak }} + {\text{eijk}}$$

The original article [1] has been corrected.
