# Supplementary material for: MicroBVS: Dirichlet-tree multinomial regression models with Bayesian variable selection - an R package
Source: BMC Bioinformatics. 2020 Jul 22;21:301. doi: 10.1186/s12859-020-03640-0 (PMC7359232; doi:10.1186/s12859-020-03640-0)
Supplement: Supplementary file 1 — Additional file 1 Supplementary Material for MicroBVS: Dirichlet-Tree Multinomial Regression Models with Bayesian Variable Selection - an R Package. [file 12859_2020_3640_MOESM1_ESM.pdf]

SOFTWARE

Supplementary Material for MicroBVS: Dirichlet-Tree Multinomial Regression Models with Bayesian Variable Selection - an R Package

Matthew D. Koslovsky* and Marina Vannucci

*Correspondence:
mkoslovsky12@gmail.com
Department of Statistics, Rice University, Houston, TX, USA

Technical Details

In order to accommodate a tree-like structure among compositional data, the Multinomial distribution is decomposed into the product of Multinomial distributions for each of the sub-trees in the tree, and the conjugate Dirichlet-tree prior is applied. Specifically, let tree T have K leaf nodes and V internal nodes. Let C_v represent the set of child nodes for each individual node $v \in V$. For each subject, the branch probability between parent node v and child node c is represented as $p_{i,vc}$, where $\sum_{c=1}^{|C_v|} p_{i,vc} = 1$ and $|C_v|$ is the number of child nodes of v. Under this parameterization, we assume that $y_{i,v} = (y_{i,v1}, \ldots, y_{i,vC})'$ follows a Multinomial$(\dot{y}_{i,v}, p_{i,v})$, where $p_{i,v} = \{p_{i,vc}, c \in C_v\}$. We assume a Dirichlet$(\gamma_{i,v})$ prior for each $p_{i,v}$, where $\gamma_{i,v} = (\gamma_{i,vc} > 0, \forall c \in C_v)$. Integrating the $p_{i,v}$ out, we model $\dot{y}_{i,v}$ with a Dirichlet-Multinomial$(\gamma_{i,v})$ and take the product of the v Dirichlet-Multinomial (DM) models for each sub-tree, to obtain the Dirichlet-tree Multinomial (DTM) distribution as

$$\prod_{v \in V} \frac{\Gamma(\sum_{c \in C_v} y_{i,vc} + 1)\Gamma(\sum_{c \in C_v} \gamma_{i,vc})}{\Gamma(\sum_{c \in C_v} y_{i,vc} + \sum_{c \in C_v} \gamma_{i,vc})} \times \prod_{c \in C_v} \frac{\Gamma(y_{i,vc} + \gamma_{i,vc})}{\Gamma(y_{i,vc} + 1)\Gamma(\gamma_{i,vc})},$$

where Γ represents the gamma function. The generalized DM model, as well as the DM model, are special cases of the DTM class of models [1]. Specifically, the generalized DM model can be represented as a DTM with a binary cascading tree (i.e., at each level of the tree, the right-most branch splits into two), and the DM can be represented with a tree containing only one root node and K leaf nodes.

Similar to [2], covariate effects can be incorporated into the model using a log-linear regression framework. Specifically, we set $\lambda_{i,vc} = \log(\gamma_{i,vc})$ and assume

$$\lambda_{i,vc} = \alpha_{vc} + \boldsymbol{x}_i' \varphi_{vcp},$$

where $\boldsymbol{x}_i = (x_{i,1}, \ldots, x_{i,P})'$ represents a set of measurements on P covariates and $\boldsymbol{\varphi}_{vc} = (\varphi_{vc1}, \ldots, \varphi_{vcP})'$. We assume the intercept terms α_{vc} follow a $N(0, \sigma^2_{vc})$, where σ^2_{vc} are set large to impose vague priors on α_{vc}.

Variable Selection Priors

Under the parameterization presented in above, the number of potential models to choose from when performing variable selection grows quickly, even for small

covariate spaces. For example, $P = 10$ covariates and just $K = 2$ compositional taxa results in over a million potential models. To reduce the dimension of the model, we employ multivariate variable selection spike-and-slab priors that identify dietary covariates that are associated with each compositional taxon [3], as opposed to spike-and-slab constructions that select variables as relevant to either all or none of the responses [4]. Here, we assume that the covariates' inclusion in the model is characterized by a latent, $K \times P$-dimensional inclusion matrix $\boldsymbol{\zeta}$. With this formulation, $\zeta_{kp} = 1$ indicates that covariate p is associated with compositional taxon k and 0 otherwise. The prior for φ_{kp} given ζ_{kp} follows a mixture of a normal distribution and a Dirac-delta function at zero, δ_0, and is commonly referred to as the spike-and-slab prior. Specifically,

$$\varphi_{kp}|\zeta_{kp}, r_k^2 \sim \zeta_{kp} \cdot N(0, r_k^2) + (1 - \zeta_{kp}) \cdot \delta_0(\varphi_{kp}), \tag{1}$$

where r_k^2 is set large to impose a vague prior for the regression coefficients in the case of covariate inclusion.

The prior probability of inclusion for each covariate, ζ_{kp}, can take various forms, which can incorporate different levels of sparsity into the model and can accommodate relations between covariates. Commonly a Beta-Binomial distribution is used. With this prior, we assume each ζ_{kp} follows a Bernoulli distribution

$$p(\zeta_{kp}|\omega_{kp}) = \omega_{kp}^{\zeta_{kp}}(1 - \omega_{kp})^{1-\zeta_{kp}},$$

and further impose a Beta prior on $\omega_{kp} \sim \text{Beta}(a, b)$. By integrating out ω_{kp}, we obtain

$$p(\zeta_{kp}) = \frac{\text{Beta}(\zeta_{kp} + a, 1 - \zeta_{kp} + b)}{\text{Beta}(a, b)},$$

where the hyperparameters a and b can be set to impose various levels of sparsity in the model. In practice, [2] suggest using a weakly-informative prior probability of inclusion by setting $a + b = 2$, where the prior expected mean value $m = a/(a + b)$. Thus, setting $a = 0.2$ and $b = 1.8$ is interpreted as a prior belief that 10% of the covariates will be selected. A non-informative prior is assumed by setting $a = b = 1$ (i.e., $m = 0.50$). See [2] for a detailed sensitivity analysis regarding hyperparmeter specification for DM regression models, which may be applied to our extensions in the following sections. Lastly, we assume the intercept terms α_k follow a $N(0, \sigma_k^2)$, where σ_k^2 are set large to impose vague priors.

Network Priors

Oftentimes researchers are interested in incorporating prior information for the probability of inclusion of a covariate based on known relations with other covariates. For example, when covariates are chosen as KEGG pathways or gene expression levels, a network of covariate interactions may be known based on biological information [5, 6]. This graphical structure can be incorporated into the model via Markov random field (MRF) priors, which are parameterized to increase a covariate's inclusion probability if neighboring covariates in the graph are included.

MRFs are undirected graphical models for random variables whose distribution follows Markovian properties.

To incorporate this structure into the variable selection framework, the prior probability of inclusion for each covariate is set according to the given relations between covariates $\boldsymbol{x}$. Specifically, we place a MRF prior on ζ_k that increases the probability of inclusion for a covariate if covariates in its neighborhood in the graph are also included. Given the graph G, an adjacency matrix that represents the relations between covariates, the prior probability of inclusion for indicators ζ_k follows

$$p(\zeta_k|G) \propto \exp(a_G \mathbf{1}'\zeta_k + b_G \zeta_k' G \zeta_k),$$

where $\mathbf{1}$ is a P-dimensional vector of 1s and a_G and b_G control the global probability of inclusion and the influence of neighbors' inclusion on a covariate's inclusion, respectively. Previous studies have demonstrated how small increments in b_G can drastically increase the number of covariates included in the model [5, 6]. Li and Zhang [5] provide a detailed description of how to select a value for b_G. Note that if there is no structure within the covariate space, the prior probabilities of inclusion reduce to independent Bernoulli$(\exp(a_G)/(1+\exp(a_G)))$.

Determining G

Oftentimes, G is set based on known relations between covariates. However in more exploratory scenarios when less is known about relations, the network structure can be learned. Efficient sampling algorithms for learning the structure of high-dimensional data with Gaussian graphical models [7] have allowed researchers to embed them into Bayesian variable selection models that simultaneously perform variable selection while learning the relations between covariates [8].

Let $X \sim MVN(\mathbf{0}, \boldsymbol{\Omega})$, where $\boldsymbol{\Omega} = \Sigma^{-1}$ is a $P \times P$ precision matrix. Following [7], we assume a hierarchical prior that models conditional dependence between covariates through edge detection in an undirected graph. Let graph G contain P nodes, corresponding to the set of potential covariates in the model. Let $g_{st} \in \{0,1\}$ represents a latent inclusion indicator for an edge between nodes s and t, for $s < t$. The inclusion of edge g_{st} corresponds to $\omega_{st} \neq 0$, where ω_{st}, $1 \leq s < t \leq P$, are the off-diagonal elements of $\boldsymbol{\Omega}$. The prior distribution for $\boldsymbol{\Omega}$ is the product of P exponential distributions for diagonal components and $P(P-1)/2$ mixtures of normals for off-diagonal components of the precision matrix. Specifically, $\mathrm{p}(\boldsymbol{\Omega}|G, v_0, v_1, \theta) = \{C(G, v_0, v_1, \theta)\}^{-1} \prod_{s<t} N(w_{st}|0, v_{st}^2) \prod_s \mathrm{Exp}\,(w_{ss}|\theta/2)\, I_{\{\boldsymbol{\Omega} \in M^+\}}$, where $C(G, v_0, v_1, \theta)$ is a normalizing constant, $v_0 > 0$ is set small to push ω_{st} to zero for excluded edges, $v_1 > 0$ is set large to allow ω_{st} to be freely estimated for included edges, and $I_{\{\boldsymbol{\Omega} \in M^+\}}$ is an indicator function that constrains $\boldsymbol{\Omega}$ to be a symmetric-positive definite matrix. The prior for the edge inclusion indicator g_{st} follows $\mathrm{p}(\mathrm{G}, \mathrm{v}_0, v_1, \theta, \pi) = \{C(v_0, v_1, \theta, \pi)\}^{-1} C(G, v_0, v_1, \theta) \prod_{s<t} \left\{\pi^{g_{st}}(1-\pi)^{1-g_{st}}\right\}$, where $C(v_0, v_1, \theta, \pi)$ is a normalizing constant and π represents the prior probability of inclusion for an edge.

Posterior Inference

In Bayesian inference, the posterior distribution is proportional to the product of the likelihood of the data and the prior distributions for the parameters. We implement a

Metropolis-Hastings algorithm within a Gibbs sampler. Assuming a Beta-Binomial prior probability of inclusion, the parameter space is described as $\Phi = \{\boldsymbol{\alpha}, \boldsymbol{\varphi}, \boldsymbol{\zeta}\}$, and the posterior distribution is

$$p(\Phi|\boldsymbol{Y}, \boldsymbol{x}) \propto f(\boldsymbol{Y}|\boldsymbol{\alpha}, \boldsymbol{\varphi}, \boldsymbol{\zeta}, \boldsymbol{x})p(\boldsymbol{\alpha})p(\boldsymbol{\varphi}|\boldsymbol{\zeta})p(\boldsymbol{\zeta}).$$

We use a two-step update approach to sample regression coefficients and inclusion indicators for covariates, following [9].

A generic iteration of the MCMC algorithm is described as follows:

- Update each α_{vc} - Metropolis step with random walk proposal from $\alpha'_{vc} \sim N(\alpha_{vc}, 0.50)$. Accept proposal with probability

$$\min\left\{\frac{f(\boldsymbol{Y}|\boldsymbol{\alpha}', \boldsymbol{\varphi}, \boldsymbol{\zeta}, \boldsymbol{x})p(\alpha'_{vc})}{f(\boldsymbol{Y}|\boldsymbol{\alpha}, \boldsymbol{\varphi}, \boldsymbol{\zeta}, \boldsymbol{x})p(\alpha_{vc})}, 1\right\}.$$

- Jointly update a ζ_{vcp} and φ_{vcp} -
  - *Between-Model Step* - Randomly select a ζ_{vcp} term. z@
    * Add: If the covariate is currently excluded ($\zeta_{vcp} = 0$), change it to $\zeta'_{vcp} = 1$. Then sample a $\varphi'_{vcp} \sim N(\varphi_{vcp}, 0.50)$. Accept proposal with probability

$$\min\left\{\frac{f(\boldsymbol{Y}|\boldsymbol{\alpha}, \boldsymbol{\varphi}', \boldsymbol{\zeta}', \boldsymbol{x})p(\varphi'_{vcp}|\zeta'_{vcp})p(\zeta'_{vc})}{f(\boldsymbol{Y}|\boldsymbol{\alpha}, \boldsymbol{\varphi}, \boldsymbol{\zeta}, \boldsymbol{x})p(\zeta_{vc})}, 1\right\}.$$

    * Delete: If the covariate is currently included ($\zeta_{vcp} = 1$), change it to $\zeta'_{vcp} = 0$ and set $\varphi'_{vcp} = 0$. Accept proposal with probability

$$\min\left\{\frac{f(\boldsymbol{Y}|\boldsymbol{\alpha}, \boldsymbol{\varphi}', \boldsymbol{\zeta}', \boldsymbol{x})p(\zeta'_{vc})}{f(\boldsymbol{Y}|\boldsymbol{\alpha}, \boldsymbol{\varphi}, \boldsymbol{\zeta}, \boldsymbol{x})p(\varphi_{vcp}|\zeta_{vcp})p(\zeta_{vc})}, 1\right\}.$$

  - *Within-Model Step* - Propose a $\varphi'_{jp} \sim N(\varphi_{jp}, 0.50)$ for each covariate currently selected in the model ($\zeta_{vcp} = 1$). Accept each proposal with probability

$$\min\left\{\frac{p(\boldsymbol{Y}|\boldsymbol{\alpha}, \boldsymbol{\varphi}', \boldsymbol{\zeta}, \boldsymbol{x})p(\varphi'_{vcp}|\zeta_{vcp})}{p(\boldsymbol{Y}|\boldsymbol{\alpha}, \boldsymbol{\varphi}, \boldsymbol{\zeta}, \boldsymbol{x})p(\varphi_{vcp}|\zeta_{vcp})}, 1\right\}.$$

To include a known graphical structure and impose a MRF prior for selection, the algorithm simply replaces $p(\zeta)$ with $p(\zeta|G)$. If the relational structure between the covariates is unknown, the posterior distribution of the model is re-defined as

$$p(\Phi|\boldsymbol{Y}, \boldsymbol{X}) \propto f(\boldsymbol{Y}|\boldsymbol{\alpha}, \boldsymbol{\varphi}, \boldsymbol{\zeta}, \boldsymbol{X})f(\boldsymbol{X}|\boldsymbol{\Omega})p(\boldsymbol{\alpha})p(\boldsymbol{\Omega}|G)p(\boldsymbol{\varphi}|\boldsymbol{\zeta})p(\boldsymbol{\zeta}|G)p(G),$$

where $\Phi = \{\boldsymbol{\alpha}, \boldsymbol{\varphi}, \boldsymbol{\zeta}, \boldsymbol{\Omega}, G\}$. Note that this parameterization treats the covariates $\boldsymbol{X}$ as random and not fixed. For implementation, the MCMC algorithm requires two additional steps to simultaneously learn the graphical relations. In our implementation, we update $\boldsymbol{\Omega}$ and G following the approach outlined in [7].

The algorithm is initiated at a set of arbitrary parameter values and then used to generate samples of the posterior distribution. After burn-in, the remaining samples

are used for inference. To determine inclusion in the model, the marginal posterior probability of inclusion (MPPI) for each of the covariates is determined by taking the average of their respective inclusion indicator's MCMC samples. Note that a covariate has a unique inclusion indicator for each of the taxon. Commonly, variables are included in the model if their MPPI ≥ 0.50 [10]. Alternatively, [11] propose using a threshold based on a Bayesian false discovery rate (BFDR) to control for multiplicity.

References

1. Wang, T., Zhao, H.: A Dirichlet-tree multinomial regression model for associating dietary nutrients with gut microorganisms. Biometrics **73**(3), 792–801 (2017)
2. Wadsworth, W.D., Argiento, R., Guindani, M., Galloway-Pena, J., Shelburne, S.A., Vannucci, M.: An integrative Bayesian Dirichlet-multinomial regression model for the analysis of taxonomic abundances in microbiome data. BMC Bioinformatics **18**(1), 94 (2017)
3. Stingo, F.C., Chen, Y.A., Vannucci, M., Barrier, M., Mirkes, P.E.: A bayesian graphical modeling approach to microrna regulatory network inference. The Annals of Applied Statistics **4**(4), 2024–2048 (2010)
4. Brown, P.J., Vannucci, M., Fearn, T.: Multivariate Bayesian variable selection and prediction. Journal of the Royal Statistical Society: Series B (Statistical Methodology) **60**(3), 627–641 (1998)
5. Li, F., Zhang, N.R.: Bayesian variable selection in structured high-dimensional covariate spaces with applications in genomics. Journal of the American statistical association **105**(491), 1202–1214 (2010)
6. Stingo, F.C., Chen, Y.A., Tadesse, M.G., Vannucci, M.: Incorporating biological information into linear models: A Bayesian approach to the selection of pathways and genes. The Annals of Applied Statistics **5**(3) (2011)
7. Wang, H., *et al.*: Scaling it up: Stochastic search structure learning in graphical models. Bayesian Analysis **10**(2), 351–377 (2015)
8. Peterson, C.B., Stingo, F.C., Vannucci, M.: Joint bayesian variable and graph selection for regression models with network-structured predictors. Statistics in Medicine **35**(7), 1017–1031 (2016)
9. Savitsky, T., Vannucci, M., Sha, N.: Variable selection for nonparametric gaussian process priors: Models and computational strategies. Statistical science: a review journal of the Institute of Mathematical Statistics **26**(1), 130–149 (2011)
10. Barbieri, M.M., Berger, J.O., *et al.*: Optimal predictive model selection. The Annals of Statistics **32**(3), 870–897 (2004)
11. Newton, M.A., Noueiry, A., Sarkar, D., Ahlquist, P.: Detecting differential gene expression with a semiparametric hierarchical mixture method. Biostatistics **5**(2), 155–176 (2004)
